# The Action of Topical Remedies on Inflamed Dentine

**Published:** 1856-10

**Authors:** Geo. Watt


### DENTAL REGISTER OF THE WEST.

OCTOBER, 1856.

### Original Essays and Communications.

### THE ACTION OF TOPICAL REMEDIES ON INFLAMED DENTINE,

BY GEORGE WATT.

*Read at the American Dental Convention, August 7th,* 1856.

Local remedies, generally the main dependence, and often the only resort of the dental surgeon, in the treatment of the diseases intrusted to his care, should be carefully considered and thoroughly understood by him. It is true that constitutional remedies are frequently required in the treatment of dental disease; and it is probable that we are often inclined to neglect the general, and rely on the topical treatment; yet, in inflammation of the dentine, from the nature of the tissue involved, it is evident that, in a majority of cases, the local is the treatment indicated.

The reader will please bear in mind that we now have nothing to say on irritable or exposed pulps, but that our remarks apply only to cases of exalted sensibility of the dentine.

For the sake of clearness, let us bear in mind that dentine, like other bony tissue, is composed of animal matter and earthy salts; that it possesses vitality; that its various constituent parts are capable of uniting chemically with other substances, and of undergoing chemical decomposition; and that it is sustained in its present state of existence by the combined influences of affinity, cohesion, and vital force.

Many difficulties lie in the way of reliable experiments, in relation to the reactions which take place when chemical agents are brought in contact with dentine. It will not do to depend on their action on the tooth out of the mouth; for then the chemical affinity is not counteracted by vitality. Neither can we rely on the reactions set up by them with gelatin, albumen, or the various earthy salts of which dentine is composed; for, in that case, affinity is counteracted neither by vitality nor by the cohesion of the tooth. But, though we cannot learn all that we wish to know from these experiments, still we can learn much that is both interesting and useful.

By making due allowance, in each case, for the circumstances present to modify affinity, and by comparing the results with those we witness in the living teeth, as, from time to time, these agents are applied, we can arrive at conclusions much more reliable, as a basis for practice, than can be derived from any series of empirical experiments, even though it extends to millions of cases and claims the accumulated light of generations. Additional light may also be obtained by noticing the topical action of these remedies on the soft parts, and on the fluids of the system.

Local or topical remedies produce mechanical, chemical, and vital effects. It is of the chemical that we propose principally to treat. An agent whose action depends on affinity, even though not capable of producing a chemical change in the tissue to which it is applied, may still be properly ranked as a chemical remedy.

The action of a chemical remedy depends on the strength of its affinity for any or all of the constituents of the tissue on which it acts; on the texture of that tissue; on the nature of the resulting compounds, and on the presence or absence of modifying circumstances. Other things being equal, combination takes place with far more energy between liquids, than between a solid and a liquid, or two solids. This is because the solid state prevents that closeness of contact necessary to an energetic manifestation of affinity, which acts only at insensible distances, and because the cohesion of the solid prevents that mobility of its ultimate particles which is necessary to combination. A soft or porous body is, therefore, attacked by an agent incapable of acting on a more dense one of the same composition. The relative action of a strong acid on chalk and marble will illustrate the point in consideration. The more dense the dentine, then, the greater is its capability of resisting chemical action.

The tendency of any substance having a strong affinity for organic matter, when in contact with living tissue, is to overcome the vitality of the part, and unite with one or more of its constituents. Substances capable of producing these changes are called *caustics*, or *escharotics*. The destruction of vitality in one part produces a change in the vital actions of the surrounding parts, usually resulting in inflammation, or, at least, in exalted vitality. The whole action of these agents is, therefore, a *chemico-vital* process. The vital action thus aroused is in proportion to the amount of the disturbance, and to the vitality of the tissue. In the dentine, therefore, the chemical will generally predominate over the vital action; although in some of the soft parts, possessing abundant vitality, the reverse is often the case. The vital force may, in some cases, be able to prevent the escharotic action of these remedies, if they are diluted, or if the energy of their affinity for organic matter be in any way diminished. An immediate chemical change may be thus prevented, and the life of the part preserved; but the vital action is disturbed and altered. The active force is here still supposed to be affinity, and the effect is termed irritation. A prolonged application of weak chemical agents, however, will finally produce slight changes in the composition of the tissues, without causing the death of the altered parts. A caustic may, accordingly, become either an irritant or an astringent, by dilution, or any other means by which the energy of its affinity for organized matter is lessened.

Water, albumen, fibrin, gelatin and the calcareous salts are the constituents of dentine, on which the various chemical remedies in use exert their action. And, it may be added, that agents inducing caries expend the force of their affinities on the same constituents. For example, nitric acid coagulates the albumen, dissolves the phosphate of lime, and decomposes the carbonate—in short, it acts chemically on every constituent of dentine, and its action ceases only when it is neutralized by the various combinations which take place. The same is true of hydrochloric acid, but not to the same extent; for a dilute solution of it spends its force almost entirely on the calcareous portion of the tooth, leaving the gelatinous portion behind.

The caustic alkalies act by their affinities for water, albumen, fibrin, and gelatin, which are very powerful; but, having little or no affinity for the earthy portion of the tooth, their action on dentine is less violent than that of most acids. The calcareous matter predominating so greatly, shields the gelatinous portions, to a great extent, from the action of such agents. The action of these agents on solid albumen, &c., is more like ordinary solution than definite chemical combination.

The character and texture of the compounds, resulting from the union of escharotics, or astringents, with any or all of the constituents of a living tissue, will, if carefully observed, do much to enable us to select the proper remedy for a particular case. The points important to be noticed are, whether the resulting compound is soluble, or insoluble; and if insoluble, whether it be permanent, or liable to rapid decomposition. On this point, experiments out of the mouth, with medicinal agents, on the various organic constituents of dentine, will give much important information. As an illustration of what is meant, let us compare the action of a caustic alkali with that of creosote, or a kindred substance. It is well known that the alkalies dissolve and hold in solution solid albumen, fibrin, and gelatin. Now, if one of these be applied to dentine, the animal portion of its substance is dissolved and removed, leaving the surface, with its increased vitality, amounting to irritation or inflammation, exposed to the action of the atmosphere, the fluids of the mouth, and any irritating agent that may be brought in contact with it.

It is evident, then, that the exalted sensibility of dentine is not likely to be relieved by such agents, unless applied in quantity sufficient to dissolve the gelatin to such depth that the undissolved calcareous matter becomes a protecting surface.

On the other hand, the application of creosote, or a substance possessed of similar chemical properties, is followed by a union of the agent with the organic constituents of the dentine, resulting in the formation of an insoluble compound, retained in place by its mechanical connection with the calcareous portion of the tooth, forming a perfect protection to the parts beneath, which, by excluding all foreign substances, permits the newly aroused vital actions to perform their proper functions, in restoring the parts to health.

Another point to be noticed, in this connection, is the fact that the soluble compounds thus formed in dental cavities are liable to be absorbed, and thus produce the specific effect of the remedy on the whole body of the dentine, as well as on the pulp, while the absorption of the insoluble is, in the nature of things, simply impossible. And this leads to the important distinction, already alluded to, between the insoluble compounds which are permanent, and those which are readily decomposed. The former, as already stated, cannot be absorbed, while the latter often are. Those formed by the action of tannin, or chloride of zinc, represent the one class —those from arsenious acid, or chloride of mercury, the other.

It is important to bear in mind that the agent possessing the strongest affinity for the constituents of dentine is not necessarily the most energetic in its action. Tannin manifests a powerful affinity for albumen, fibrin, &c.; but, as the resulting compound is insoluble and permanent, the very energy of the affinity prevents the full force of the remedy, by the almost instantaneous formation of a protective layer, which guards the subjacent parts against the further action of the drug. This remedy is, therefore, practically mild in its operation, its action being necessarily confined to a thin superficial layer, whether the quantity used be great or small; yet, to the extent of its combination with the organic constituents of the tissue, vitality is as thoroughly overcome as it could be by the actual cautery.

On the other hand, chloride of zinc, though manifesting less active affinity for the organic constituents of dentine, is, at the same time, a far more active remedy. This arises from several causes. It manifests a powerful affinity for water, and in removing this from the dentine, it prepares the way for its own admission, and, at the same time is dissolved, and is thus in a better condition for exerting its other affinities, which are in no degree impaired by its union with the water. The compounds resulting from its union with albumen, &c., are more soluble than those formed with tannin, and consequently present a feebler barrier to the action of the remaining portions of the drug. The combination taking place less rapidly affords time for the agent to penetrate the dentine to a greater depth. And besides, the chloride, in contact with organic matter, is gradually decomposed, and thus a limited portion of its chlorine is liberated, and unites with the calcareous elements of the tooth.

In the language of Pereira,—"The components of the living part, when combined with any of these substances (which form with them insoluble compounds), are less susceptible of decay and decomposition than previously. Hence they are unfitted for the principal property which appertains to their vital condition, viz: that of suffering and effecting transformation." It is on this principle that these remedies exert an antiseptic influence; and it follows that the agent which produces the most permanent insoluble compound with the constituents of the tissue, is the most reliable local antiseptic; while those producing compounds more soluble, and less permanent, often exert a more extended antiseptic influence on large masses of organic matter, simply because they pass into its substance more readily, and to a greater depth. It is well known that the antiseptic influence of tannic acid on the dead skin is perfect, leather being merely tannate of gelatin, while that of arsenious acid, or chloride of mercury, is imperfect. At the same time, by injecting the vessels of the dead subject with either of the latter substances, putrefaction is for a long time prevented, while an injection of tannin could preserve but little, if any, more than the vascular tissues, on account of its inability to pass out of the principal vessels, owing to the nature of the compound it forms with their organic constituents.

Some of the remedies which exert an antiseptic influence act, at the same time, as disinfectants. The chlorides which undergo partial decomposition, when in contact with organic matter, are the most striking examples of this class. The liberated chlorine decomposes the putrefactive agents, and effectually destroys the attendant fetor. The carious portions remaining in the cavity, therefore, cease, for the time, to be a source of irritation. This, added to the protecting power of the decomposed layer, which is insoluble, explains the fact that when chloride of zinc or terchloride of gold is used on a diseased surface, whether of the soft or bony tissues, the parts beneath the decomposed surface are found healthier than before the application, and more so than when an escharotic of a different class is applied.

By bearing in mind the laws of combination, and the doctrine of chemical equivalents, we might be led to conclude that, when the affinities are equal, the energies of chemical remedies are inversely as their combining proportions; but, that the rule may hold good, not only the affinities, but all the modifying circumstances must be equal. It is certainly true, that if two agents manifest equal affinities for the same constituents of dentine, and the resulting compounds are of the same texture, their modus operandi must be the same. In such cases, and only in such, is the activity of the remedies inversely as their equivalents. The equivalent of creosote is 94, that of tannin 212, and the practical activity of the two remedies bears some proportion to these numbers; but if we notice the modus operandi of creosote, we will see that its practical energy is much increased by its capability of dissolving fats and other organic substances, thus removing these obstacles to that intimate contact necessary to chemical action. On the other hand, we have seen that but a limited quantity of tannin can be made to act on any surface of organized structure. It follows, therefore, that the practical activity of the one is greater, and that of the other less than is indicated by their equivalents.

It is hoped that the reader already appreciates the fact that there are important differences, both in the modes and in the results of the action of chemical remedies for inflamed dentine. Due discrimination, and accurate judgment are, therefore, necessary in selecting the remedies for any given case. The state of the general system, the physiological structure, and the pathological condition of the tooth must be understood before it is possible to know the treatment indicated. Then, the remedy most likely to fulfill the indication is to be selected, and, without a knowledge of the modus operandi of each of those from which the choice is to be made, the selection is, at best, but mere guesswork.

That we may have a clear understanding of this point, let us briefly notice two or three different conditions of the dentine, which may require treatment. Take, for example, a tooth in which the whole body of dentine is inflamed, or at least has exalted sensibility. Now, it is evident, that cutting out the sensitive portion is not the plan for this case; for layer after layer may be removed, till the central cavity is reached, and the sensibility may be increased during the entire operation. The patient is therefore tortured to the limit of endurance, while nothing is gained. Nor is the sensibility immediately relieved by the influence of a remedy whose action is limited to the surface; for when the vitality of a superficial layer is thus destroyed, an attempt to remove it demonstrates the fact that the sensibility of the subjacent parts is in no degree diminished, but rather increased. The sensibility in this case, however, may be promptly and totally subdued by an escharotic, which, from its modus operandi, is capable of exerting its action on the deep-seated, as well as on the superficial parts; and those who wish to resort to this mode, will find arsenious acid all they could desire. This course, however, seems to us much like that of the woman who strangled her bed-ridden husband, by way of "helping to ease his misery."

The course we prefer, in such a case, is the application of an agent which produces an insoluble and permanent compound with the gelatinous portion of the tooth, thus preserving the parts beneath from the influence of irritating agents, till the case has time to terminate in resolution. This protection should be rendered more complete by the insertion of a soft filling into the cavity; and the termination may be much facilitated by constitutional treatment.

In other cases the exalted sensibility may be confined to the surface of the carious cavity. In such, agents which exert a prompt, yet superficial action will accomplish all that is desired. The selection will depend somewhat on the circumstances of the case, and will be better understood, after the consideration of the individual remedies.

In some cases the inflammation is confined to one or more minute points in the cavity. Then the sharp cutting instrument is often sufficient to overcome the difficulty. When it is not, the treatment suggested for the preceding case may be resorted to.

In considering the individual remedies for inflamed dentine, we do not propose to notice all that have been, or that may be used with advantage, but only a number sufficient to accomplish the various actions indicated, and to meet the ordinary demands of practice. We will not attempt a classification; for in the limited number under consideration nothing could be gained by it.

The first that we will notice is

TANNIN, OR TANNIC ACID.—Tannic acid is the active principle of vegetable astringents, and is found more abundant in nut-galls than in any other product. It manifests strong affinities. It is soluble in water and alcohol, and slightly so in ether. It unites with albumen, fibrin, and gelatin, forming with them insoluble tannates. It thus enables us to detect gelatin when dissolved in several thousand times its weight of water. Its medicinal action is almost necessarily topical; for the promptness of its action on, and the insolubility of its compounds with albuminous substances, prevents its admission into the general circulation. And this is the sole reason that the vegetable astringents are comparatively mild and innocuous in their action; for a single grain of tannin, if conveyed directly into the blood, would cause instant death.

The action of tannin on dentine has been already explained. Either its watery, or alcoholic solution may be used; the latter is the most convenient, in some respects, as the former suffers decomposition by the absorption of oxygen from the atmosphere.

CREOSOTE, OR CARBOLIC ACID.—This agent produces its caustic effects by its affinity for albumen and gelatin; and its antiseptic influence arises from the fact that it forms with these substances insoluble compounds.

The creosote of former years was obtained from wood-tar; and in some respects it differs from that in present use, which is prepared from coal-tar. The latter is the genuine carbolic acid. Its medicinal effects are the same as those of the wood-tar creosote, while it is not so unpleasant. It dissolves freely in alcohol and ether, and sparingly in water. Its action may therefore be modified by dilution.

The action of creosote on dentine has been already explained; and, from its modus operandi, it is evident that the popular opinion that it promotes the decay of the teeth is an error. Its other uses do not fall within the range of this paper.

NITRATE OF SILVER.—This salt is a powerful caustic, whether applied to the soft parts or to the bony tissues. Its action is somewhat complex. Dr. Turner imputes its escharotic power to the action of the nitric acid which is liberated by the decomposition of the salt in contact with organic matter. This, however, explains but a part of the process; for the salt seems to have a strong affinity for albumen, and unites with it without undergoing decomposition, in the proportion, according to Lassaigne, of 84.5 of albumen to 15.5 of the salt. This compound is soluble in a solution of nitrate of silver, or of chloride of sodium.

When the nitrate is applied to the skin, the immediate result is a whitish mark, caused by the union of the salt with the albumen of the cuticle. This soon becomes black, by the decomposition of the salt and the reduction of the oxyd of silver. It is evident, then, that for each atom of silver set free, an equivalent of nitric acid is liberated. With these facts before us, we will be able to understand its action on dentine.

Let us, then, bear in mind that we have an agent here which acts promptly on the gelatinous portion of the tooth, destroying its vitality to the extent of the combination which takes place; and that by the decomposition of a part of the salt, and the consequent liberation of a part of its acid, it acts also with energy on the calcareous portion.

The compound formed by the nitrate with the organic constituents of the tooth is insoluble, except in a few substances, and therefore protects the subjacent parts, as mentioned in speaking of tannin. The precipitation of the reduced oxyd on the surface affords some additional protection.

The insolubility of the compound above-mentioned, prevents the absorption of the nitrate by the dentine, and renders its action necessarily superficial. It is not true, then, that its application endangers the pulp, unless the intervening portion of dentine be so thin that it is all required in the chemical union which takes place between it and the remedy; but it is true that its judicious application adds to the safety of the pulp, by relieving the inflammation of the dentine, which might, otherwise, be extended to it.

When the nitrate is neutralized, by an equivalent of the constituents of the dentine uniting with it, no farther chemical action can ensue; but it should be borne in mind that the compound formed by its union with the organic portion of the tooth is soluble in a solution of the nitrate. By applying it in too great a quantity, or too frequently, there may be a greater loss of substance than is desirable or at all necessary; for, as long as free nitrate remains in solution in the cavity, the insoluble compound is not precipitated, and the surface is, therefore, exposed to its continued action. This constitutes a great practical difference between its action and that of tannin; for we have seen that, however much of the latter may be present, but a small quantity of it has the opportunity of producing chemical action.

The compound of the nitrate with the organic constituents of the tooth is soluble also in chloride of sodium; hence, when the fluids of the mouth abound in this salt, the nitrate does not afford that protection to the subjacent dentine which may be obtained by some other escharotics; and in any mouth, the protection is inefficient, if the surface be exposed to contact with food seasoned with the chloride.

In view of the above facts, we prefer to use the nitrate in the solid state; and when this is not practicable, we use a concentrated solution in small quantity, in preference to repeated applications of a dilute one.

In consideration of the caustic energy of the nitrate, as compared with that of arsenic,—knowing that the latter is often absorbed and destroys the vitality of the tooth, many fear that the pulp is alike endangered from its use. From the remarks already made, we think it is plain that their fears are groundless. We will add, however, that all authorities we have been able to consult, agree that it is not absorbed, even when applied to the soft parts, but that its action is *necessarily* confined to the surface. And farther: in acute cases of poisoning, by its internal use, there is seldom—perhaps never—any evidence of its absorption.

The subjacent portion of dentine is generally less healthy after the application of the nitrate than after the use of a proper chloride; but, if properly used, the destruction of dentine will be less with the former than with the latter.

With a clear understanding of the modus operandi of the nitrate, the practitioner will be at no loss in regard to the cases demanding its use. It acts to a greater depth than tannin, or creosote, but not so deep as chloride of zinc, nor does it produce as much pain. Of its action on the soft tissues, we have nothing to say in this paper.

CHLORIDE OF ZINC.—The chloride of zinc is, perhaps, more frequently applied to dentine than any other caustic. From its modus operandi, it exerts an antiseptic and disinfectant, as well as escharotic influence. Its principal action is on the animal portion of the dentine; yet, as already seen, a part of it is decomposed, and the liberated chlorine may act on the calcareous salts. As its caustic power depends, in part, on its affinity for water, it is milder in solution than in substance; and its action is, consequently, more superficial and less painful. It is soluble in water, alcohol, ether, and chloroform. The ethereal and chloroformal solutions produce far less pain than the chloride in substance. This might be readily expected—its affinity for water being thus overcome, it exerts but a part of its caustic power. Its union with the gelatinous portion of the tooth is also more prompt when thus dissolved; and this may, in part, explain the diminution of pain arising from its application; as the ethereal solution of terchloride of gold, which is yet more prompt, causes still less pain. On the same principle, the actual cautery, when very hot, causes less pain and irritation than when of a lower temperature. The ether or chloroform may, however, act, directly, in lessening the pain, by local anasthæsia.

In using the chloride, or any other active caustic, it is important to remember the exalted vitality that follows its use. Practitioners are sometimes disappointed in its action, by either delaying the operation too long or beginning too soon after its application. The former, we apprehend, is the most frequent error. They wait till the exalted vitality commences, but not till it subsides. Now, we regard this as an important point; and it is difficult to lay down definite rules respecting it. It is evident that in the teeth of young persons, and especially in those where the animal matter greatly predominates, the vitality will be more promptly aroused than in those of the opposite texture, and, at the same time, the vital change will be greater. Now, if the exalted sensibility be confined to a thin, superficial layer, it may be almost instantly subdued by the application of the ethereal or chloroformal solution, and the cavity may be excavated before the vitality of the subjacent portion is excited. But if the operation be delayed till the reaction is established, the tooth is often found in a worse condition for excavating than before the application, and a further postponement becomes necessary.

The remarks made on absorption, when speaking of the nitrate of silver, apply with equal force here. There is not the least possible danger from this source—there can be none, even when the chloride is applied to the soft parts.

TERCHLORIDE OF GOLD.—Of this substance, we have used only the ethereal solution. It acts with great promptness on dentine, forming an insoluble compound with the gelatinous portions; and, by its decomposition, and the consequent liberation of chlorine, it acts also on the calcareous salts. On account of its promptness, neither the pain nor the disturbance of the subjacent parts is great. It is, consequently, very convenient when the exalted sensibility is superficial. The greatest inconvenience connected with its use is its great liability to decomposition. By exposure to air or light, the gold is precipitated in the metallic form. With due care, however, it can be preserved a long time, and it is easily prepared. There is, probably, no danger in its use from absorption; but a more extended series of experiments and observations are required to warrant a positive statement on this point.

ARSENIOUS ACID.—The modus operandi of arsenious acid is involved in great obscurity. In regard to its topical action, Professor Bache says: "Arsenious acid, when it produces the death of a part, does not act, strictly speaking, as an escharotic. It destroys the vitality of the organized structure, and its decomposition is the consequence. The true escharotic acts chemically, producing the decomposition of the part to which it is applied: a state incompatible with life." Pereira says: "Though employed as a caustic, yet the nature of its chemical influence on the animal tissues is unknown. Hence, it is termed by some a dynamical caustic." Its escharotic power certainly bears no proportion to its destruction of vitality. That it forms definite compounds with some of the constituents of living tissues, is highly probable; yet, if so, they appear to be readily and rapidly decomposed, by which means the acid is again free to effect similar results with the subjacent parts of the tissue.

Nearly all authorities agree that the topical application of arsenic is liable to be followed by constitutional effects. All dentists admit that the tooth pulp may be destroyed by it, through a wall of dentine of considerable thickness. In general, they maintain that, to accomplish this, the agent must, in some way, penetrate the substance of the dentine. Now, as the dentine is endowed with but feeble vitality, it is evident that its life is destroyed by the agent, to the extent that it penetrates it. Consequently, the vitality of a great portion of the dentine may be lost, by the use of the remedy, even when the pulp is not reached. The exalted sensibility of dentine is, then, subdued by this agent, more by its *vital* than by its chemical effects.

The leading argument in favor of the use of arsenic for inflamed dentine is its reliability. Well, it is reliable—and no mistake. Whether the augmented sensibility be confined to a spot, a superficial layer, or extend to the whole body of the dentine, it is alike efficient. It subdues the sensibility in these cases, just as effectually as, when sweetened with syrup, or diluted with water, it overcomes the vitality of rats and cockroaches. The exalted vitality, incident to ordinary escharotic action, is not likely to annoy the operator who uses this remedy; for all such reaction is soon subdued by it. As the despot suppresses the earliest uprisings for liberty beneath his iron heel, so this tyrant drug will not tolerate, in the subjacent dentine, even the slightest attempt at rebellion.

The most soluble preparations of arsenic are the most energetic; and the quickness with which it acts is in proportion to the absorbing powers of the part. It is sometimes extensively used as a topical application, without inducing constitutional effects; and, in other cases, the constitutional symptoms are alarming, from the local use of a very minute quantity. The whole weight of authority, we think, demonstrates the fact that the pulp is never safe when it is applied to a carious cavity of a tooth; and, as inflamed dentine may be otherwise relieved, that it should never be applied to a tooth, unless the extirpation of the pulp is indicated.

